# ORF8 and Health Complications of COVID-19 in Down Syndrome Patients

**DOI:** 10.3389/fgene.2022.830426

**Published:** 2022-02-09

**Authors:** Antonio Bensussen, Antonio Valcarcel, Elena R. Álvarez-Buylla, José Díaz

**Affiliations:** ^1^ Laboratorio de Dinámica de Redes Genéticas, Centro de Investigación en Dinámica Celular, Universidad Autónoma del Estado de Morelos, Cuernavaca, Mexico; ^2^ Centro de Ciencias de la Complejidad (C3), Universidad Nacional Autónoma de México, Ciudad de México, Mexico; ^3^ Laboratorio de Genética Molecular, Epigenética, Desarrollo y Evolución de Plantas, Instituto de Ecología, Universidad Nacional Autónoma de México, Ciudad de México, Mexico

**Keywords:** SARS-CoV-2, COVID-19, ORF8, Down syndrome, Cardiac damage

This manuscript intends to be a “letter to the editor” with some complementary commentaries to the manuscript by Valcarcel et al. (2021). In that work, ORF8 was characterized as a notable SARS-CoV-2 protein able to downregulate the function of MHC-I (Zhang et al., 2021) and which shares structural similarities with human immunoglobulins (including interleukins) that can eventually produce immune dysregulation (Valcarcel et al., 2021). It is not still clear if all COVID-19 patients are equally susceptible to this ORF8-mediated immune dysregulation, but Down syndrome (Ds) patients with COVID-19 have more health complications, such as cardiac diseases, and higher rate of mortality than the general population, especially in those over 40 years old (Hüls et al., 2021). Ds is an important comorbidity since these patients have an extra copy of the *TMPRSS2* gene, which probably produces enhanced levels of the transmembrane TMPRSS2 protease for S protein priming, facilitating the SARS-CoV-2 infection of the target cells (Hoffmann et al., 2020; De Toma and Dierssen, 2021).

Therefore, we proposed a minimal mathematical model of the effect of the extra copy of TMPRSS2 on ORF8 production and persistence in the infected cells (Figure 1A), which reasonably fits with the experimental data reported in literature. According to the model results, we found that systemic levels of ORF8 are considerably higher and persists up to 40 days in patients with Ds (Figures 1B, C) in contrast with patients without Ds. These results support our hypothesis that the *high susceptibility of people with Ds to be infected by SARS-CoV-2 is a consequence of the overproduction of* TMPRSS2, *which produces high systemic levels of* ORF8 *with the subsequent immune dysregulation, lung inflammatory effects, and cardiac damage that worsen the disease* (Espinosa, 2020).

**FIGURE 1:**
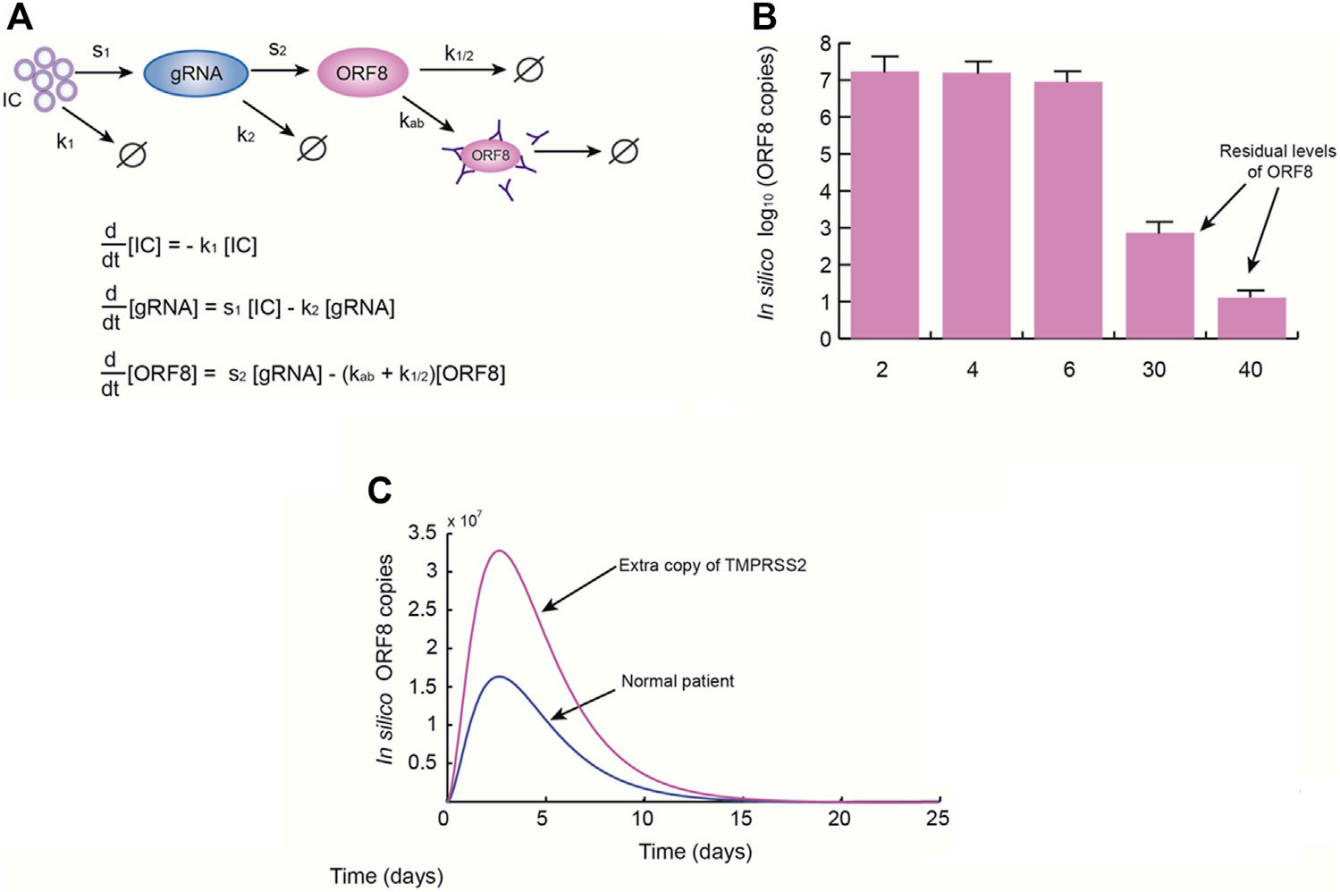
Down syndrome patients have higher levels of ORF8 during SARS-COV-2 infection. **(A)** Once the SARS-CoV-2 ACE2-Spike complexes enter the cells (*IC*), the production of genomic RNA (gRNA) originates ORF8 antigens by several editing steps. These copies of ORF8 may be naturally degraded or eliminated by antibodies. **(A)** The simplified model of ORF8 production used in this work. In the model *k*_1_ = 0.4 days^−1^, *s*_1_ = 100 days^−1^, *k*_2_ = 1.3 days^−1^, *s*_2_ = 20 days^−1^, *k*_*ab*_ = 0.3 days^−1^, and *k*_1/2_ = 0.5 days^−1^. **(B)** Mean levels of ORF8 obtained from the model simulations at 2, 4, 6, 30, and 40 days. These results suggest that ORF8 persists even if the patient is discharged 15 days after viral onset. **(C)** Effect of the presence of an extra copy of TMPRSS2. These simulations show that an extra copy of *TMPRSS2* gene is able to dramatically increase systemic levels of ORF8, which implies that Down syndrome patients are more susceptible to medical complications produced by ORF8.

Additional consequences of the overproduction of ORF8 in Ds patients with COVID-19 are as follows: 1) the several structural similarities of this viral protein with the nitric oxide synthase can alter the serum concentrations of NO, reducing the protective function of this gas against arrhythmias (Burger and Feng, 2011); 2) ORF8 can also be an important factor to aggravate the cytokine storm due its high degree of structural mimicry with immunoglobulins and their receptors (Valcarcel et al., 2021), with the subsequent small protoembolic events that cause a cardiovascular damage similar to that of older Ds patients never infected with Covid-19 (Colvin and Yeager, 2017; De Toma and Dierssen, 2020). 3) Taking into consideration that chromosome 21 also harbors multiple genes involved in the immune response, and their overexpression induces the dysregulation of interleukins IL-10, IL-22, and IL-26 prior to infection (De Weerd and Nguyen, 2020), the presence of high levels of ORF8 could also be an important factor to aggravate the cytokine storm in Ds patients with COVID-19.

However, it is necessary to do more theoretical, experimental, and clinical research to elucidate the precise role of ORF8 in the immune dysregulation, lung inflammatory effects, and cardiac damage in this group of patients.

## Appendix

The model we used in this work takes into account the levels of the Spike-ACE2-TMPRSS2 complex (*IC*) that allows the internalization of the SARS-CoV-2 genomic RNA (*gRNA*) and the systemic levels of ORF8. To model ORF8 systemic levels, we considered the half-life of the protein (Can et al., 2020) as well as the effect of neutralizing antibodies against ORF8 (Hachim et al., 2020) (Figure 1A). Next, we used *ex vivo* data obtained from COVID-19 patients (Bar-On et al., 2020; Hachim et al., 2020; Peng et al., 2020; Wölfel et al., 2020) to estimate parameters and to calibrate the model congruently with previous observations (Peng et al., 2020). The model was numerically solved using the Runge–Kutta 4-5 method and MATLAB software.
